# Phenotypic Heterogeneity in Glaucoma: The Systemic and Vascular Landscape Behind Functional Loss

**DOI:** 10.1155/mi/7874763

**Published:** 2026-01-30

**Authors:** José Enrique Muñoz de Escalona Rojas, José Luis García Serrano, Pablo Navarro Martínez

**Affiliations:** ^1^ Department of Ophthalmology (Glaucoma Unit), Hospital Universitario Clínico San Cecilio, Granada, Andalusia, Spain, husc.es; ^2^ Department of Ophthalmology (Glaucoma Unit), Hospital Universitario del Vinalopó, Alicante, Elche, Valencian Community, Spain

**Keywords:** central corneal thickness, normal-tension glaucoma, primary open-angle glaucoma, structural equation modeling, systemic risk factors

## Abstract

**Objective:**

To analyze the direct and indirect associations between structural, functional, and systemic variables in patients with primary open‐angle glaucoma (POAG), normal‐tension glaucoma (NTG), and controls using structural equation modeling (SEM).

**Methods:**

A cross‐sectional observational study was conducted including 156 participants: 55 with POAG, 49 with NTG, and 52 age‐ and sex‐matched controls. Clinical variables included intraocular pressure (IOP), central corneal thickness (CCT), vertical cup‐to‐disc ratio (VCDR), and visual‐field mean deviation (MD). Systemic variables comprised age and cardiovascular risk factors (hypertension, diabetes, and dyslipidemia). SEM was applied to assess direct and indirect effects on the diagnosis of glaucoma.

**Results:**

The model showed a good overall fit (*χ* ^2^(9) = 15.968; *p* = 0.068; *χ* ^2^/df = 1.774; CFI = 0.972; RMSEA = 0.071) and explained 46.0% of the variance in glaucoma diagnosis (*R* ^2^ = 0.460). The most influential predictors were VCDR (*β* = 0.40), age (*β* = 0.14), and cardiovascular risk factors (*β* = 0.19). A significant negative correlation was observed between CCT and MD (*r* = –0.29), indicating greater functional damage in eyes with thinner corneas. An inverse association between cardiovascular risk burden and IOP was also identified (*r* = –0.18).

**Conclusions:**

Our findings support the hypothesis of distinct glaucomatous phenotypes, including a vascular subtype with lower IOP and altered perfusion, potentially influenced by systemic comorbidities and chronic treatments. Reduced CCT was also confirmed as an independent marker of advanced functional loss. SEM helps disentangle complex mechanisms and may inform personalized therapeutic strategies, particularly in NTG.

## 1. Introduction

Glaucoma is a chronic, progressive optic neuropathy and one of the leading causes of irreversible blindness worldwide. Its global prevalence in adults aged 40–80 years is estimated at ~3.5%, with a particularly high burden in Asia and Africa [1]. Primary open‐angle glaucoma (POAG) is the most common form, with an estimated prevalence of 2.4% in the adult population, corresponding to about 68 million people affected in 2020 [2]. This number is projected to rise to more than 110 million by 2040, driven mainly by population aging and the persistence of systemic risk factors [1].

POAG is characterized by an open anterior chamber angle and elevated intraocular pressure (IOP), whereas normal‐tension glaucoma (NTG) presents glaucomatous optic neuropathy and visual‐field loss despite IOP remaining within the statistically normal range [3]. While elevated IOP is the main modifiable risk factor in POAG, its role in NTG is more limited. In the latter, additional mechanisms have been identified, such as ocular hypoperfusion, impaired vascular autoregulation, and greater structural susceptibility of the lamina cribrosa [4]. Although both subtypes share similar anatomical and functional features, they differ in risk profiles and epidemiological distribution [3, 4].

Several ocular structural and functional parameters have been established as predictors of glaucoma onset and progression. Central corneal thickness (CCT) is one of the most relevant structural indicators. The OHTS showed that thinner corneas are associated with a higher risk of conversion to glaucoma in patients with ocular hypertension [5], and recent systematic reviews have confirmed that CCT and corneal biomechanics are independent risk factors [6]. In NTG, a thin cornea may also lead to underestimation of true IOP or reflect lower biomechanical resistance of the optic nerve head [4].

The vertical cup‐to‐disc ratio (C/D) is another well‐established structural biomarker. Greater cupping has been associated with an increased risk of conversion and glaucomatous progression, especially when accompanied by Drance hemorrhages in NTG [6, 7]. Functionally, visual‐field mean deviation (MD) is the standard parameter for quantifying functional loss and monitoring progression. An MD decline greater than 1.5 dB/year has been correlated with faster visual deterioration and a worse prognosis [8].

Beyond ocular factors, systemic factors also play a key role in glaucoma pathogenesis. Older age is a universal risk factor affecting both POAG and NTG, likely due to age‐related structural and vascular changes [5]. In POAG, systemic arterial hypertension has been modestly associated with increased risk, possibly through mechanisms such as increased episcleral venous pressure or impaired autoregulation [6]. In contrast, in NTG, systemic hypotension—particularly nocturnal dips in diastolic pressure—has been consistently linked to disease progression [4]. Diabetes mellitus and dyslipidemia show variable associations across populations, although recent meta‐analyses support their inclusion as systemic factors contributing to POAG risk [6].

Given the multifactorial nature of glaucoma and the interdependence of multiple clinical, structural, and systemic variables, conventional statistical models may be insufficient to capture its complexity. In this context, structural equation modeling (SEM) has become a powerful analytical tool that allows assessment of direct and indirect relationships between observed variables and latent constructs. SEM can simultaneously estimate multiple causal pathways, account for measurement error, and model factors not directly observable, such as ocular perfusion [4]. Recent studies have successfully applied this methodology to explore how IOP, vascular dysfunction, and optic nerve structure interact in the genesis and progression of glaucoma [4, 8].

## 2. Hypotheses and Study Rationale

Based on the available evidence, we propose the following hypotheses:

• The combination of ocular structural parameters, functional measures, and systemic factors exerts an additive and interdependent effect on glaucoma risk.
• SEM can disentangle these complex interactions and identify latent factors that contribute to glaucomatous damage beyond the direct effect of IOP.

The aim of this study was to analyze the direct and indirect associations between structural, functional, and systemic variables in patients with POAG, NTG, and controls using an SEM framework. This approach seeks to provide a more integrated understanding of glaucoma, optimize risk stratification, and contribute to the development of personalized management strategies.

## 3. Materials and Methods

### 3.1. Study Design and Ethical Approval

A cross‐sectional observational study was conducted at the Department of Ophthalmology of Hospital Universitario Clínico San Cecilio (Granada, Spain). The study protocol adhered to the principles of the Declaration of Helsinki and was approved by the Andalusian Biomedical Research Ethics Committee (registration number: ES12345GL; approval date: May 6, 2025). All participants provided written informed consent.

### 3.2. Participants

A total of 156 consecutive patients attending specialized glaucoma clinics were included. Inclusion criteria were age ≥40 years and a confirmed diagnosis of POAG or NTG. POAG was defined by characteristic structural and functional findings with documented untreated IOP >21 mmHg on at least two separate occasions (*n* = 55). NTG was defined by glaucomatous optic neuropathy with corresponding visual‐field defects and untreated IOP ≤21 mmHg (*n* = 49). An age‐ and sex‐matched control group without glaucoma from the same health area was also included (*n* = 52).

Exclusion criteria included a history of ocular trauma, uveitis, secondary glaucoma (e.g., steroid‐induced), advanced retinal disease (such as diabetic retinopathy), non‐glaucomatous optic neuropathies, unreliable visual fields, incomplete clinical records, or prior ocular surgery (except uncomplicated phacoemulsification).

### 3.3. Clinical Assessment

All participants underwent a standardized ophthalmologic evaluation, including:

• Best‐corrected visual acuity.
• Slit‐lamp biomicroscopy.
• Goldmann applanation tonometry.
• Gonioscopy.
• Dilated fundus examination.
• Optic nerve optical coherence tomography (OCT).
• Standard automated perimetry (SAP), 24‐2 SITA Standard strategy (Humphrey Field Analyzer, Carl Zeiss Meditec).
• CCT measured by ultrasound pachymetry.

Systemic data, including age, hypertension, diabetes mellitus, and dyslipidemia, were collected from medical records.

### 3.4. Variables and Statistical Analysis

**Figure 1:**
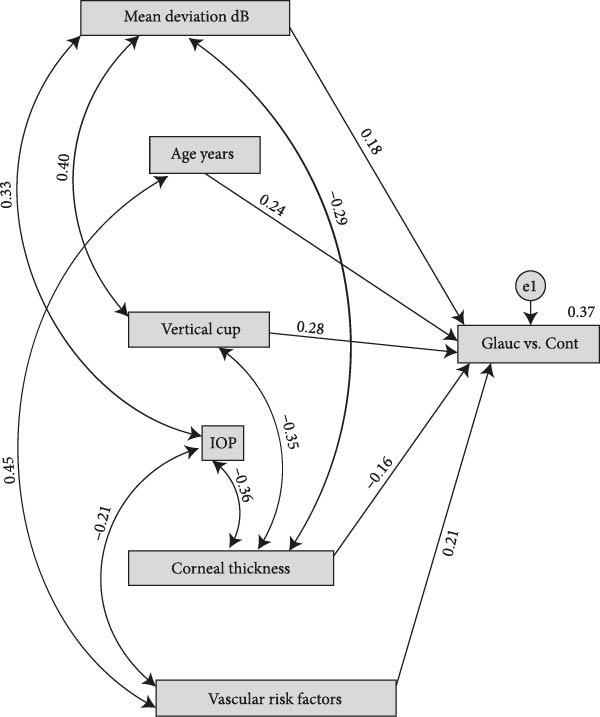
Variables included in the structural equation model (SEM). The model includes age (years), intraocular pressure (IOP, mmHg), central corneal thickness (CCT, μm), vertical cup‐to‐disc ratio (VCDR), visual‐field mean deviation (MD, dB), and the presence of cardiovascular risk factors (hypertension, diabetes, and/or dyslipidemia).

Variables included in the structural equation model (SEM) were age (years), IOP (mmHg), CCT (μm), vertical cup‐to‐disc ratio (VCDR), visual‐field mean deviation (MD, dB), and the presence of cardiovascular risk factors (hypertension, diabetes, and/or dyslipidemia) (Figure 1).

For interpretability, visual‐field mean deviation (MD, dB) was entered into the SEM as a positive‐scale index of functional loss (MD inv = –MD), such that higher values indicate greater visual‐field defect and values close to 0 indicate no or minimal functional loss.

In the SEM, glaucoma (including both NTG and POAG) was treated as the dependent variable. All analyses were performed with a 5% significance level. Only bivariate associations that were statistically significant and showed a correlation greater than 0.3 were included in the model. The following model‐fit criteria were used as reference: chi‐square (*χ* ^2^, *p*  > 0.05), comparative fit index (CFI >0.90), root mean square error of approximation (RMSEA <0.08), standardized root mean square residual (SRMR <0.08), goodness‐of‐fit index (GFI >0.90), and Tucker–Lewis index (TLI >0.90). These criteria indicate the closeness of the model to the observed data.

For SEM purposes, a binary variable “GlaucvsCont” (0 = control; 1 = glaucoma) was created by grouping patients with POAG and NTG. This decision was made to (i) increase statistical power and model stability and (ii) identify common factors associated with glaucoma diagnosis (glaucoma vs. control) across both subtypes, consistent with the objective of exploring shared patterns of phenotypic heterogeneity.

The model allowed evaluation of direct and indirect associations among variables and their relationship with the glaucoma diagnosis. Standardized regression coefficients (*β*) were calculated to quantify effect sizes. Statistical analyses were performed using SPSS (IBM Corp., Armonk, NY) and IBM SPSS AMOS (Analysis of Moment Structures).

### 3.5. Sample Size Considerations

A sample size of 156 participants was considered adequate based on recommendations suggesting a minimum of 5–10 cases per estimated parameter. This size provides acceptable statistical power to detect significant associations and reliable estimation of model parameters and fit indices [9]. The three study groups had clearly defined diagnoses from the outset, which reduced diagnostic variability and enabled more focused estimation. In our case, the minimum subgroup size was 49 patients, with statistical power *β* = 0.20, provided that the SEM parameters showed good model fit [9].

## 4. Results

### 4.1. Structural Model Fit

The model showed an adequate overall fit: *χ* ^2^(9) = 15.97, *p* = 0.068; *χ* ^2^/df = 1.77; GFI = 0.972; AGFI = 0.914; CFI = 0.974; IFI = 0.975; TLI = 0.939. The approximation error was reasonable (RMSEA = 0.071; 90% CI 0.000–0.126; pclose = 0.23). Information criteria favored the proposed model compared with alternative models (AIC = 53.97; BIC = 111.92), and the expected cross‐validation index was better (ECVI = 0.348 vs. 0.361 and 1.947). Nevertheless, the wide 90% CI for RMSEA (0.000–0.126) indicates limited precision for this index; therefore, fit should be interpreted jointly with the remaining metrics (CFI, TLI, GFI/AGFI, and *χ* ^2^/df), which together support an overall acceptable model fit (Table 1).

**Table 1:** Model fit indices for the structural equation model.

Index	Observed value	Recommended threshold	Meets criterion?
*χ* ^2^ (df = 9)	15.968 (*p* = 0.068)	*p* > 0.05	Yes ✔️
CMIN/DF	1.774	<3.00	Yes ✔️
GFI	0.972	>0.95	Yes ✔️
AGFI	0.914	>0.90	Yes ✔️
RMSEA	0.071	<0.08	Yes ✔️
pclose (RMSEA)	0.239	>0.05	Yes ✔️
CFI	0.974	>0.95	Yes ✔️

*Note:* Values indicate good model fit when recommended thresholds are met.

Abbreviations: *χ* ^2^, Chi‐square; AGFI, Adjusted Goodness‐of‐Fit Index; CFI, Comparative Fit Index; CMIN/DF, normed chi‐square; df, degrees of freedom; GFI, Goodness‐of‐Fit Index; pclose, probability associated with RMSEA; RMSEA, Root Mean Square Error of Approximation.

### 4.2. General Structural Model

Figure 1 shows the SEM model with standardized regression coefficients. Six independent variables were evaluated: visual‐field MD, VCDR, central corneal thickness (CCT), IOP, age, and cardiovascular risk factors. All predictors showed significant relationships with the latent variable “Glaucoma vs Control.”

### 4.3. Regression Weights

In the structural model, the probability/propensity for glaucoma was significantly associated with higher VCDR (*β* = 0.405; *p*  < 0.001), lower CCT (*β* = −0.188; *p* = 0.004), higher cardiovascular risk burden (*β* = 0.188; *p* = 0.005), older age (β = 0.144; *p* = 0.032), and greater visual‐field deficit (MD_inv = ‐MD; *β* = 0.181; *p* = 0.006). Among predictors, VCDR showed the largest standardized effect (Table 2).

**Table 2:** Standardized regression weights in the structural equation model.

Predictor → GlaucvsCont	B (SE)	C.R.	*p*	*β*	Brief interpretation
Mean deviation (dB; MD_inv = –MD)	0.014 (0.005)	2.724	0.006	0.181	Positive, significant effect: greater visual‐field deficit (MD_inv) → higher probability/propensity for glaucoma.

Age (years)	0.006 (0.003)	2.139	0.032	0.144	Older age → higher probability/propensity for glaucoma (small‐to‐moderate effect).

VCDR (Vertical cup)	0.760 (0.131)	5.795	<0.001	0.405	Strongest effect: greater vertical cupping → higher probability/propensity for glaucoma.

CCT (μm)	−0.002 (0.001)	−2.872	0.004	−0.188	Thinner cornea → higher probability/propensity for glaucoma.

Cardiovascular risk factors	0.180 (0.064)	2.806	0.005	0.188	Presence/increased vascular risk burden → higher probability/propensity for glaucoma.

*Note: p* value: statistical significance. Standardized estimates reflect the magnitude and direction of the relationship between each predictor and the glaucoma diagnosis in the model. MD_inv is derived by multiplying the original MD by −1 (MD_inv = –MD), such that higher values indicate greater functional loss.

Abbreviation: C.R., critical ratio.

### 4.4. Covariances and Correlations Between Variables

The covariance analysis showed significant relationships among multiple pairs of exogenous variables. Among the most relevant associations, a negative correlation was found between CCT and visual‐field mean deviation (MD) (*r* = –0.29), suggesting that thinner corneas are associated with greater functional deterioration. In addition, a negative correlation was identified between cardiovascular risk factors and IOP (*r* = –0.18), as well as between CCT and IOP (*r* = –0.35), an unexpected association according to prior literature. This latter negative correlation between CCT and IOP may be explained by the mixed nature of the sample, which includes NTG patients and patients receiving IOP‐lowering medical or surgical treatment, thereby altering the usual physiological relationship between CCT and IOP. For example, NTG often presents with reduced CCT and low IOP ranges, and treatment may have artificially lowered IOP in patients with thicker corneas, thus reversing the typical relationship (Table 3).

**Table 3:** Correlations between exogenous variables.

Variable 1	Variable 2	Correlation (*r*)
Vertical cup‐to‐disc ratio	Central corneal thickness	−0.42
Central corneal thickness	Intraocular pressure (IOP)	−0.35
Mean deviation (dB)	Central corneal thickness	−0.29
Mean deviation (dB)	IOP	0.28
Cardiovascular risk factors	IOP	−0.18
Cardiovascular risk factors	Age (years)	0.48
Mean deviation (dB)	Vertical cup‐to‐disc ratio	0.45

*Note:* Pearson correlation coefficients (*r*) showing linear associations among the exogenous variables included in the SEM. All correlations shown are statistically significant (*p*  < 0.05). Clarification: in these correlations, MD corresponds to MD_inv ( = –MD); therefore, higher values represent greater functional loss.

### 4.5. Variances and Coefficient of Determination (*R* ^2^)

The variances of observed variables were significant (*p*  < 0.001): MD = 35.529; age = 122.054; VCDR = 0.058; CCT = 1,200.424; cardiovascular risk factors = 0.224; IOP = 21.084. The model explained 46.2% of the variance in the endogenous variable (*R* ^2^ = 0.462), with an estimated residual variance of 0.110 (Table 4).

**Table 4:** Estimated variances of observed variables and coefficient of determination.

Variable (unit)	Variance (Est.)	Standard error	C.R.	*p*
Mean deviation (dB)	35.529	3.970	8.950	<0.001
Age (years)	122.054	13.864	8.803	<0.001
Vertical cupping (VCDR, unitless)	0.058	0.007	8.803	<0.001
CCT (μm)	1,200.424	133.329	9.003	<0.001
Cardiovascular risk factors (index)	0.224	0.025	8.893	<0.001
IOP (mmHg)	21.084	2.372	8.889	<0.001

*Note: p* value: statistical significance. *R* ^2^ represents the proportion of variance explained in glaucoma diagnosis (glaucoma vs control) by the structural model. *R* ^2^ for GlaucvsCont = 0.462 (Squared Multiple Correlations) → the set of predictors explains 46.2% of the variance in glaucoma/control status.

Abbreviation: C.R., critical ratio.

Therefore, all observed‐variable variances are significantly >0 (C.R. ≈8.8–9.0; *p*  < 0.001), indicating sufficient and well‐estimated variability in the model.

The residual variance of GlaucvsCont is low (e1 = 0.110), consistent with a moderate‐to‐high *R* ^2^ (46%), supporting the explanatory capacity of the model with the included predictors.

## 5. Discussion

In our study, we identified two relevant findings related to chronic glaucoma. First, we found a significant inverse correlation between the number of cardiovascular risk factors (arterial hypertension, diabetes mellitus, and dyslipidemia) and intraocular pressure (IOP), with a correlation coefficient of *r* = –0.18. This result, obtained in a population comprising patients with both POAG and NTG, contrasts with previous studies in the literature, which typically report a positive relationship between cardiovascular risk factors and mild IOP elevation or glaucoma risk [10, 11].

Several epidemiological studies have shown that metabolic factors such as arterial hypertension, diabetes mellitus, and dyslipidemia—often grouped within metabolic syndrome—are typically associated with modest increases in IOP. Lee et al. [12] reported an average increase of 0.33 mmHg in IOP for each additional cardiovascular risk factor present. Other population‐based studies also reported significantly higher IOP in patients with POAG and metabolic syndrome compared with individuals without these factors [13, 14].

Nevertheless, the relationship between cardiovascular risk factors, IOP, and glaucomatous neuropathy appears more complex and bidirectional. Recent studies have described different phenotypes within chronic glaucoma, particularly in NTG, distinguishing a metabolic phenotype and a vascular phenotype. The latter is characterized by peripheral vascular dysregulation, nocturnal hypotension, and ischemic phenomena [15, 16].

**Figure 2:**
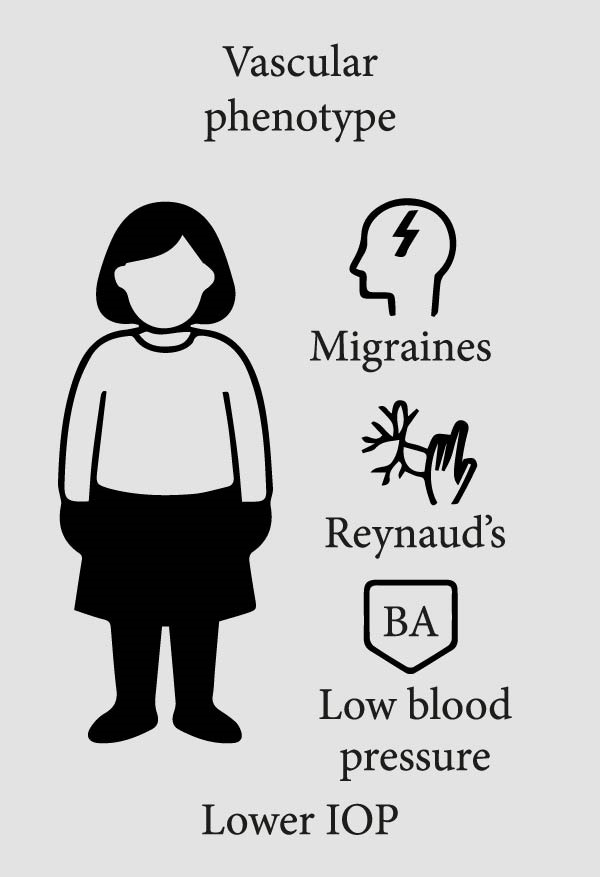
Vascular glaucoma phenotype with lower intraocular pressure. Diagram illustrating a glaucomatous phenotype characterized by lower intraocular pressure (IOP), commonly associated with normal‐tension glaucoma and influenced by vascular dysregulation and cardiovascular risk factors.

**Figure 3:**
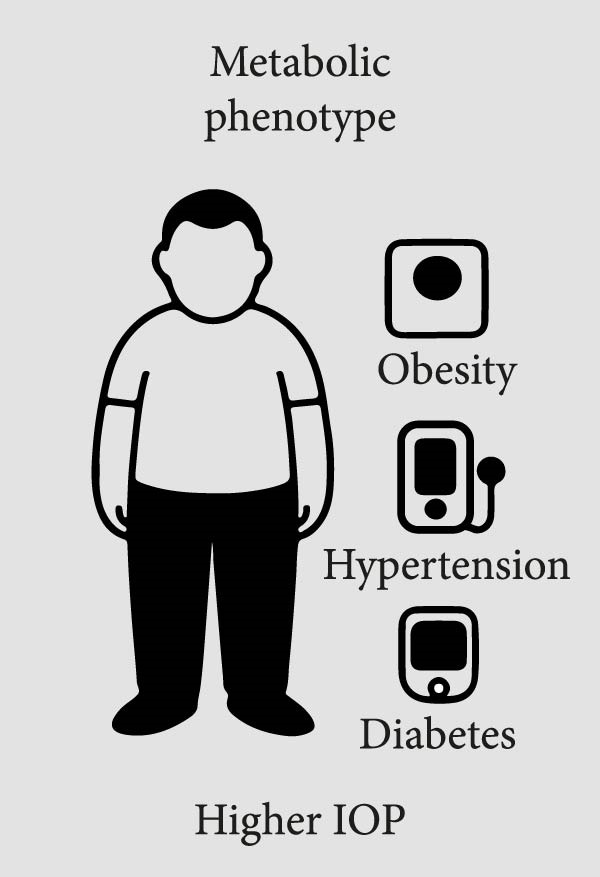
Metabolic glaucoma phenotype with higher intraocular pressure. Diagram illustrating a glaucomatous phenotype characterized by higher intraocular pressure (IOP), more frequently associated with primary open‐angle glaucoma and a metabolic risk profile.

Patients with the vascular phenotype typically have lower IOP (Figure 2), whereas individuals with a metabolic profile require relatively higher pressures to develop glaucomatous damage (Figure 3) [15, 17]. In this context, our observation of an inverse correlation could indicate a relative predominance of the vascular phenotype in our population. On the other hand, it should be noted that our glaucoma group includes NTG patients and POAG patients treated with topical medication and/or surgery; thus, pressures in this group are lower, and the relationship with IOP as a variable is not significant. The inverse correlation between cardiovascular risk factors and IOP is also supported by chronic microvascular changes in the group with cardiovascular risk factors. Moreover, chronic administration of systemic medications such as beta‐blockers, metformin, or statins could reduce aqueous humor production and contribute to lower IOP; in our sample, more than 85% of patients with cardiovascular risk factors were taking at least one of these medications [18, 19].

In this context, we believe that the inverse correlation between cardiovascular risk burden and IOP identified in our SEM should be interpreted primarily as the expression of a “treated cardiovascular phenotype,” i.e., a subgroup of patients in whom IOP is already modulated by systemic medications with potential IOP‐lowering effects, rather than as a “pure” untreated vascular phenotype. In other words, this finding may represent, at least in part, a treatment‐related statistical artifact (systemic drugs that lower IOP) rather than a direct manifestation of the underlying disease biology. The available evidence regarding the effect of these systemic drugs on IOP remains limited and, in some aspects, controversial, but the high prevalence of their use in our cohort necessitates caution when interpreting the observed associations between IOP and cardiovascular risk factors.

Therefore, systemic medication should be critically considered, since drugs such as beta‐blockers, metformin, or statins could contribute to a significant IOP reduction and cause some patients initially diagnosed with NTG to actually have POAG [18, 20]. Likewise, in NTG patients with a vascular profile, the introduction of treatments that lower nocturnal blood pressure could dangerously reduce ocular perfusion pressure and exacerbate glaucomatous damage. Thus, it is crucial to carefully evaluate systemic medication and its impact on ocular and systemic dynamics in these patients [21, 22].

Methodologically, potential confounding induced by systemic medication is an important limitation of our study: we cannot precisely discriminate how much of the observed IOP reduction is due to the natural history of a vascular glaucomatous phenotype and how much is due to the hypotensive effect of systemic cardiovascular treatments. This ambiguity requires particularly cautious interpretation of our relationships among IOP, cardiovascular risk burden, and glaucoma diagnosis, and underscores the need for future studies that more exhaustively stratify systemic pharmacologic exposure and the “treated cardiovascular phenotype.”

In addition, an additional confounding factor related to the ocular status of the cohort should be highlighted. The negative correlation between central corneal thickness (CCT) and IOP (*r* = –0.35) observed in our analysis is largely explained by the inclusion of eyes already treated (both NTG and POAG) with topical IOP‐lowering therapy and/or filtering surgery, in which the recorded IOP reflects a “post‐treatment” state rather than the baseline physiological situation. Consequently, IOP measured in this cohort cannot be considered a faithful reflection of the natural physiological IOP status but rather a variable strongly conditioned by prior therapeutic interventions. This implies that all SEM pathways involving IOP (as a direct predictor or mediator) should be interpreted with extreme caution, as the reliability of these routes for inferring pathophysiological relationships is substantially limited by this treatment‐related confounding.

On the other hand, using a binary variable that combines POAG and NTG into a single group is an important limitation: this unification may attenuate or obscure subtype‐specific associations (for example, the differential role of vascular factors versus IOP). Future studies with larger sample sizes should analyze POAG and NTG separately or use multi‐group SEM to explicitly compare pathways between subtypes.

The second significant finding was the negative correlation between CCT and visual‐field MD (*r* = –0.29). CCT has been inversely associated with glaucomatous functional damage in numerous studies. Cross‐sectional evidence indicates that eyes with thinner corneas tend to present more advanced visual‐field defects, reflected in more negative MD values [23]. For example, Herndon et al. [23] (2004) found that, in POAG patients, lower CCT correlated significantly with worse visual field (more negative MD) and greater optic nerve damage at baseline. Similarly, later studies confirmed that, in bilateral glaucoma, the eye with the thinner cornea often shows a more deteriorated visual field than the fellow eye with a thicker cornea [24]. These observations extend to longitudinal designs: the Ocular Hypertension Treatment Study provided the first prospective evidence that, in subjects with ocular hypertension, a thin central cornea confers a higher risk of developing manifest glaucoma [21]. Moreover, in patients with established glaucoma, lower CCT has been shown to predict faster progression of visual loss. Kim and Chen [25] reported that, in open‐angle glaucoma, lower CCT was associated with a more rapid decline in visual field, and Chauhan et al. [26] likewise found that thinner corneas predisposed to greater deterioration in both visual field and optic disc cupping over follow‐up. Together, this clinical evidence establishes reduced CCT as a significant risk factor for both more severe glaucomatous visual damage at presentation and faster functional progression over time.

Several biomechanical and vascular hypotheses have been proposed to explain this association. From a biomechanical perspective, lower CCT may reflect reduced ocular rigidity (greater compliance), implying a more deformable lamina cribrosa that is more susceptible to mechanical stress. In other words, eyes with thin corneas may have less resistant supportive tissues (cornea/sclera), allowing greater lamina cribrosa deformation under IOP and producing more pronounced axonal damage [27]. This concept adds to the known effect of pachymetry on tonometric estimation: a thin cornea leads to underestimation of IOP with Goldmann applanation tonometry, potentially masking true ocular hypertension. Such measurement error can translate into less aggressive IOP control than indicated in patients with low CCT, indirectly contributing to greater glaucomatous damage [28]. On the other hand, vascular hypotheses suggest that a thin cornea may be a marker of optic nerve perfusion abnormalities. It has been observed that glaucomas with thinner corneas often coexist with systemic vascular risk factors, such as arterial hypotension or poor ocular blood‐flow autoregulation [29, 30]. Reduced optic nerve head perfusion—due, for example, to low ocular perfusion pressure or vasospasm—could aggravate ischemia and render neural tissue more vulnerable, enhancing functional loss even at moderate IOP. In summary, the association between reduced CCT and greater glaucomatous visual‐field damage likely reflects a combination of biomechanical factors (greater ocular deformability increasing susceptibility to pressure‐related injury) and vascular factors (compromised optic nerve head perfusion), explaining why a thin cornea is linked to a worse functional prognosis in glaucoma patients.

In conclusion, a comprehensive evaluation of glaucoma patients is essential, considering not only IOP but also systemic factors, systemic medication, and biomechanical variables such as CCT, to identify patients at risk of progression early and optimize integrated clinical management of glaucoma. In particular, our results suggest that identifying and characterizing the “treated cardiovascular phenotype” should be explicitly incorporated into clinical assessment and into the interpretation of future studies exploring the interaction among IOP, cardiovascular risk factors, and glaucomatous damage.

## Disclosure

This work has not been previously presented as an abstract at any conference or scientific meeting. The authors did not receive institutional, commercial, or private funding for this study.

## Conflicts of Interest

The authors declare no conflicts of interest.

## Funding

No funding was received for this manuscript.

## Data Availability

The data that support the findings of this study are available on request from the corresponding author. The data are not publicly available due to privacy or ethical restrictions.
